# Associations of physical activity and sedentary time with diabetic kidney disease among adults with diabetes: a multicenter population-based study

**DOI:** 10.3389/fendo.2026.1809438

**Published:** 2026-04-22

**Authors:** Jianing Liu, Shanhu Qiu, Jiali Tang, Xiaohui Guo, Zilin Sun

**Affiliations:** 1 Department of Endocrinology, Institute of Diabetes, School of Medical, Zhongda Hospital, Southeast University, Nanjing, China; 2 Department of Endocrinology, Northern Jiangsu People’s Hospital, Northern Jiangsu People’s Hospital Affiliated to Yangzhou University, Yangzhou, China; 3 Department of General Practice, Zhongda Hospital, Institute of Diabetes, School of Medicine, Southeast University, Nanjing, China; 4 Department of Endocrinology, Peking University First Hospital, Beijing, China

**Keywords:** diabetes, diabetic kidney disease, gender difference, physical activity, sedentary behavior

## Abstract

**Background:**

Increased physical activity and reduced sedentary time are associated with lower risks of diabetes. However, their association with diabetic kidney disease (DKD) is unclear. This study aimed to assess this issue in the Chinese adults with diabetes.

**Methods:**

This multicenter, cross-sectional study included adults with diabetes from 40 hospitals across 26 diverse Chinese cities. Leisure-time physical activity (LTPA), housework physical activity (HPA), occupational physical activity (OPA), and sedentary time were assessed using a validated questionnaire. DKD was defined according to the NKF-K/DOQI guidelines. Associations between activity domains and DKD were examined using multivariable logistic regression, with subgroup, interaction, and sensitivity analyses to assess robustness.

**Results:**

A total of 4,979 patients with diabetes were included. After multivariable adjustment, those meeting the guideline-recommended amount of LTPA had lower odds of DKD (OR 0.79, 95% CI 0.68–0.91 for aerobic exercise; OR 0.69, 95% CI 0.50–0.96 for resistance exercise) compared with those not meeting recommendations. However, heavier HPA and larger OPA were associated with higher odds of DKD (OR 1.51 and 1.66, respectively). Moreover, longer daily sedentary time was associated with increased odds of DKD only in women (OR 2.10), but not in men. Further stratified analysis suggested that the association between LTPA and lower odds of DKD may be modified by HPA, OPA, or sedentary time.

**Conclusions:**

Among Chinese adults with diabetes, LTPA was associated with lower odds of DKD, whereas higher occupational and household physical activity showed positive associations, underscoring the differences in domain-specific physical activity in DKD prevention.

## Introduction

Diabetic kidney disease (DKD), a prevalent microvascular complication, affects over 30% of patients with diabetes (1, 2). It represents a leading cause of end-stage renal disease (3) and is independently associated with an increased risk of cardiovascular and all-cause mortality (4, 5). Given these and considering that the number of patients living with DKD is expected to increase in the coming decades because of the increased prevalence of diabetes, identification of modifiable risk factors is therefore of clinical importance to prevent or delay the progression of DKD.

Physical activity represents a cornerstone intervention for diabetes mellitus prevention and management, with robust epidemiological evidence demonstrating its protective effects against disease development and progression (6). A prior meta-analysis showed that physical activity across all domains e.g., leisure-time physical activity (LTPA), housework physical activity (HPA), and occupational physical activity (OPA), is associated with a reduced risk of diabetes. To date, few studies have investigated the association between sedentary time and DKD. In a recent population-based analysis of 2,633 U.S. adults with diabetes from the National Health and Nutrition Examination Survey, no significant association was observed between domain-specific physical activity and DKD (7). However, the robustness of the conclusion might be weakened due to its small sample size, and it is unclear whether domain-specific physical activity show any favorable effect in lowering the odds of DKD in Chinese population. Moreover, evidence regarding the association between sedentary time and diabetic kidney disease (DKD) remains limited, despite its established links to an increased risk of diabetic retinopathy (8) and cardiovascular diseases (9). Therefore, we conducted a nationwide multicenter cross-sectional study investigating the associations of domain-specific physical activity and sedentary time with the risk of DKD in Chinese adults with diabetes.

## Methods

### Study design and participants

This multicenter, cross-sectional study used data collected from a nationally distributed network of 40 healthcare facilities—including tertiary referral centers, secondary hospitals, and community hospitals—across 26 geographically diverse Chinese cities during a standardized 4-month observation period (May-August 2014). The complete study protocol, including sampling methodology and quality control procedures, has been previously published (10). The study protocol was reviewed and approved by the Clinical Research Ethics Committee of Peking University First Hospital (approval No. 2014(725)). All participants provided informed consent at the time of enrollment.

Participants aged ≥ 18 years with a diagnosis of diabetes for at least 1 year were eligible. Of the 6,932 enrolled participants, 1,953 were excluded due to pre-diabetes diagnosis or incomplete demographic and renal data, leaving 4,979 participants included in the present analysis.

### Data collection

Trained investigators conducted face-to-face questionnaire interviews to collect data on demographic characteristics, lifestyle factors, medication history, diabetic complications, and other chronic diseases. The questionnaire’s reliability was validated by the Diabetes Care and Education Study Group of the Chinese Diabetes Society. Anthropometric and biochemical measurements included height, weight, waist and hip circumference, blood pressure, triglycerides (TG), total cholesterol (TC), high-density lipoprotein (HDL) cholesterol, low-density lipoprotein (LDL) cholesterol, fasting blood glucose (FBG), 2-hour plasma glucose (2h-PG), glycated hemoglobin (HbA1c), serum creatinine (Scr), urine protein and urinary albumin-to-creatinine ratio (UACR).

### Definitions of diabetic kidney disease and covariates

Hypertension was defined as systolic blood pressure (SBP) ≥140 mmHg and/or diastolic blood pressure (DBP) ≥ 90 mmHg, whereas hyperlipidemia was defined as total cholesterol (TC) > 4.5 mmol/L, high-density lipoprotein cholesterol (HDL-C) < 1.0 mmol/L, triglycerides (TG) > 1.5 mmol/L, and/or low-density lipoprotein cholesterol (LDL-C) > 2.6 mmol/L (11). The estimated glomerular filtration rate (eGFR) was calculated by the Xiangya equation (12), which has been validated as a more accurate method for estimating GFR in Chinese populations. DKD was ascertained based on the information from medical records, as well as the laboratory test results according to NKF-K/DOQI guidelines as any of: (1) macroalbuminuria (UACR >300 mg/g); (2) microalbuminuria (UACR 30–300 mg/g) in the presence of diabetic retinopathy; or (3) microalbuminuria in individuals with type 1 diabetes with a disease duration ≥10 years (13). Because the analysis was based on a cross-sectional survey, repeated albuminuria measurements were not available for all participants.

### Physical activity assessment and classification

Data on leisure-time physical activity (LTPA), housework physical activity (HPA), occupational physical activity (OPA), and sedentary behavior were collected by a validated questionnaire (**Supplementary Table 1**). The intensities for different physical activity forms were quantified according to the 2011 Compendium of physical activity (**Supplementary Table 2**) (13). LTPA, defined as sports or exercise performed during leisure time, was categorized into aerobic exercise (AE) and resistance exercise (RE) (14). Guideline-recommended levels for AE were defined as ≥150 minutes/week of moderate-to-vigorous activity or ≥75 minutes/week of vigorous activity, and RE as resistance training 2–3 times/week (6, 15). OPA was classified according to job titles based on associated physical demands. Students, office workers, drivers, and teachers were classified as sedentary or brain workers, representing the lowest OPA level (reference group). Homemakers, cooks, and sales staff were classified as light manual workers, while porters, construction workers, and farmers were categorized as moderate to heavy manual workers (16). HPA referred to physical activity related to housework, with ≤ 2hours/day set as the reference category. Total sedentary time was categorized as ≤ 4, 4-6, 6-8, and > 8 hours/day, representing lowest (reference), low, moderate, and highest sedentary levels, respectively.

### Statistical analysis

Continuous variables were summarized as medians (interquartile ranges) due to non-normal distributions, which were assessed using the Kolmogorov-Smirnov test for normality. Categorical variables were presented as counts and percentages. Differences between participants with and without DKD were compared using the Wilcoxon rank-sum test for continuous variables and the χ^2^ test for categorical variables.

Logistic regression models were used to estimate odds ratios (ORs) and 95% confidence intervals (CIs) for the associations between physical activity domains, sedentary behavior, and DKD risk. Two multivariable models were constructed: Model 1 adjusted for gender, age, diabetes duration, and educational level; Model 2 additionally adjusted for systolic blood pressure (SBP), body mass index (BMI), HbA1c, triglycerides (TG), high-density lipoprotein (HDL), history of hypertension, hyperlipidemia, cardiovascular diseases, and medication use.

Missing data were handled using multiple imputation by chained equations. Prior to imputation, the proportion and pattern of missing values were examined. The percentage of missingness across variables ranged from 0% to 12.69%, and the missing data patterns suggested no clear systematic structure, supporting the assumption that data were missing at random. Five imputed datasets were generated, and estimates were pooled according to Rubin’s rules. To assess the robustness of the findings, sensitivity analyses were performed using complete-case datasets, and the results were compared with those obtained from the imputed datasets. Propensity score matching (PSM) was conducted as a sensitivity analysis to assess the robustness of the findings, using greedy nearest neighbor matching with a caliper of 0.1. Covariate balance after matching was evaluated using standardized mean differences. Moreover, to evaluate the impact of domain-specific physical activity on the risk of DKD across different demographic backgrounds, we conducted subgroup analyses stratified by BMI, gender, and history of other chronic diseases. We also assessed the joint associations of leisure-time physical activity (LTPA) with occupational and housework physical activity (OPA/HPA) using stratified multivariable logistic regression models. The additional stratified analyses were performed to evaluate potential effect modification by OPA, HPA, and sedentary behavior on the LTPA-DKD association.

All analyses were conducted using R 4.2.0 (R Foundation for Statistical Computing, Vienna, Austria), Stata 15.0 (StataCorp, College Station, TX, USA), and SPSS 23.0 (SPSS Inc, Chicago, IL, USA), with two-sided P < 0.05 considered statistically significant.

## Results

### Characteristics of participants

**Table 1** summarizes the characteristics of the 4,797 included patients. The median age was 61 years, with a median diabetes duration of 8 years. Compared to patients without DKD, those with DKD exhibited a longer diabetes duration, larger waist circumference, and higher blood pressure and blood glucose levels (all P < 0.05).

**Table 1:** Baseline characteristics of participants.

Variable	DKD group	Non-DKD group	P value
Sample size (n)	N=1339	N=3640	
Age (years)	61 (53, 70)^a^	61 (52, 70)	0.306
duration of diabetes(years)	10 (5, 16)	8 (3, 14)	<0.001
Onset age (years)	50 (42, 58)	52 (43, 59)	<0.001
Male	749 (55.9%)^b^	1867 (51.3%)	0.004
History of other diseases			
Hypertension	843 (63.0%)	1868 (51.3%)	<0.001
Hyperlipidemia	255 (19.0%)	603 (16.6%)	0.040
Cardiovascular diseases	370 (27.6%)	793 (21.8%)	<0.001
Using hypoglycemic medication	1287 (96.1%)	3341 (91.8%)	<0.001
Using hypotensive medication	695 (51.9%)	1461 (40.1%)	<0.001
Weight (kg)	67.0 (59.0, 75.0)	65.5 (58.0, 74.8)	0.083
Waist circumference (cm)	90.0 (83.0, 97.0)	88.0 (81.1, 95.0)	<0.001
BMI (kg/m^2^)	24.6 (22.2, 27.1)	24.4 (22.3, 26.8)	0.129
WHR	0.93 (0.89, 0.97)	0.92 (0.88, 0.96)	<0.001
SBP (mmHg)	133 (120, 148)	129 (120, 140)	<0.001
DBP (mmHg)	80 (70, 85)	78 (70, 82)	<0.001
HbA1c (%)	8.0 (6.9, 9.9)	7.5 (6.5, 9.3)	<0.001
FBG (mmol/L)	8.0 (6.4, 10.2)	7.3 (6.3, 9.1)	<0.001
2hPG (mmol/L)	11.5 (9.0, 14.8)	10.9 (8.9, 14.1)	0.209
TC (mmol/L)	4.5 (3.7, 5.5)	4.5 (3.7, 5.2)	0.039
TG (mmol/L)	1.6 (1.1, 2.4)	1.4 (1.0, 2.1)	<0.001
LDL (mmol/L)	2.6 (2.0, 3.3)	2.6 (2.1, 3.2)	0.158
HDL (mmol/L)	1.1 (0.9, 1.3)	1.1 (0.9, 1.4)	0.005

^a^ Number (percentage %).

^b^ Median (25th percentile-75th percentile).

DKD, diabetic kidney disease; BMI, body mass index; WHR, waist circumference to hip circumference ratio; SBP, systolic blood pressure; DBP, diastolic blood pressure; HbA1c, glycated hemoglobin; FBG, fasting blood glucose; 2h-PG, 2-hour plasma glucose; TG, triglycerides; TC, total cholesterol; HDL, high-density lipoprotein; LDL, low-density lipoprotein.

### Associations of domain-specific physical activity and sedentary behavior with DKD

#### Leisure-time physical activity

After full multivariable adjustment for demographic, clinical, and biochemical covariates (Model 2), patients meeting guideline-recommended levels of LTPA had significantly lower odds of DKD (OR 0.79, 95% CI 0.68–0.91 for aerobic exercise; OR 0.69, 95% CI 0.50–0.96 for resistance exercise; **Table 2**). These findings were robust in sensitivity analyses using propensity score matching (OR 0.81, 95% CI 0.75–0.87 for aerobic exercise; OR 0.69, 95% CI 0.59–0.80 for resistance exercise; **Table 3**).

**Table 2:** The association of different physical activity domains and sedentary behavior with diabetic kidney disease.

Variable	DKD group	Non-DKD group	Crude model	Model 1	Model 2
			OR (95% CI)	OR (95% CI)	OR (95% CI)
AE meeting guideline-recommended levels					
No	406	860	1 (Ref)	1 (Ref)	1 (Ref)
Yes	910	2696	0.71 (0.62, 0.82)	0.74 (0.64, 0.85)	0.79(0.68, 0.91)
RE meeting guideline-recommended levels					
No	1222	3228	1 (Ref)	1 (Ref)	1 (Ref)
Yes	51	203	0.66 (0.48, 0.91)	0.67 (0.49, 0.93)	0.69(0.50, 0.96)
Daily housework time (hour/day)					
≤2	694	2101	1 (Ref)	1 (Ref)	1 (Ref)
2-4	182	594	0.92 (0.77, 1.12)	0.94 (0.77, 1, 14)	0.94 (0.77, 1.14)
>4	63	132	1.44 (1.06, 1.97)	1.51 (1.09, 2.08)	1.51 (1.09, 2.11)
Occupational physical activity					
Sedentary/brain worker	194	679	1 (Ref)	1 (Ref)	1 (Ref)
Light manual worker	765	2142	1.24 (1.03, 1.48)	1.08 (0.86, 1.35)	1.13 (0.90, 1.42)
Moderate/heavy manual worker	275	556	1.73 (1.40, 2.15)	1.56 (1.22, 1.98)	1.66 (1.29, 2.13)
Daily sedentary time (hour/day)					
≤4	575	1731	1 (Ref)	1 (Ref)	1 (Ref)
4-6	261	703	1.12 (0.94, 1.33)	1.13 (0.95, 1.35)	1.12 (0.93, 1.34)
6-8	137	385	1.07 (0.86, 1.33)	1.10 (0.88, 1.37)	1.05 (0.84, 1.33)
>8	92	209	1.33 (1.02, 1.72)	1.30 (0.99, 1.71)	1.16 (0.87, 1.54)

Model 1 was adjusted for gender, age, duration of diabetes, and educational level.

Model 2 was additionally adjusted for systolic blood pressure (SBP), body mass index (BMI), HbA1c, triglycerides (TG), high-density lipoprotein cholesterol (HDL-C), history of hypertension, hyperlipidemia, cardiovascular diseases, and medication usage, in addition to the variables included in Model 1.

AE, aerobic exercise; RE, resistance exercise; MI, multiple imputation; ORs, odds ratios; CIs, confidence intervals.

**Table 3:** Sensitivity analysis for the association between LTPA and DKD based on propensity score matching method.

Analysis	Groups	Number of participants	Prevalence of DKD (N, %)	OR (95% CI)	P-value
Association between AE and DKD					
Unmatched	AE meeting guideline-recommended levels	3606	910 (25.2%)	0.79 (0.68, 0.91)	<0.001
	AE not meeting guideline-recommended levels	1266	406 (32.1%)	(Ref)	
1:1 matched after MI^b^	AE meeting guideline-recommended levels	1256	346 (27.58%)	0.81 (0.75, 0.87)	<0.001
	AE not meeting guideline-recommended levels	1256	401 (31.92%)	(Ref)	
Association between RE and DKD					
Unmatched^a^	RE meeting guideline-recommended levels	254	51 (20.1%)	0.69 (0.50, 0.96)	0.01
	RE not meeting guideline-recommended levels	4450	1222 (27.5%)	(Ref)	
1:4 matched after MI^b^	RE meeting guideline-recommended levels	253	51 (20.18%)	0.69 (0.59, 0.80)	<0.001
	RE not meeting guideline-recommended levels	987	263 (26.67%)	(Ref)	

^a^ adjusted for Model 2: gender, age, duration of diabetes, educational level, SBP, BMI, HbA1c, TG, HDL, history of hypertension, hyperlipidemia, cardiovascular diseases, and medication usage;.

^b^ Balanced factors in propensity score matching: gender, age, duration of diabetes, educational level, SBP, BMI, HbA1c, TG, HDL, history of hypertension, hyperlipidemia, cardiovascular diseases, and medication usage.

AE, aerobic exercise; RE, resistance exercise; MI, multiple imputation; ORs, odds ratios; CIs, confidence intervals.

##### Occupational physical activity

More than half of participants were classified as light manual workers. Notably, a higher proportion of DKD patients engaged in moderate-to-heavy manual labor compared to non-DKD patients (20.5% vs. 15.3%, P < 0.001). Compared with sedentary/brain workers, moderate-to-heavy manual workers exhibited increased odds of DKD after full adjustment (OR 1.66, 95% CI 1.29–2.13), whereas light manual workers showed no significant association (OR 1.13, 95% CI 0.90–1.42; Model 2; **Table 2**).

##### Housework physical activity

A larger proportion of DKD patients reported performing ≥4 hours of housework daily compared to those without DKD (6.7% vs. 4.7%, P < 0.001). Relative to HPA ≤ 2 hours/day, HPA > 4 hours/day was associated with higher odds of DKD (OR 1.51, 95% CI 1.09–2.11), while HPA 2–4 hours/day showed no significant association (OR 0.94, 95% CI 0.77–1.14; Model 2; **Table 2**).

##### Sedentary time

Participants reporting sedentary time > 8 hours/day had higher odds of DKD than those with ≤4 hours/day in unadjusted analyses (OR 1.33, 95% CI 1.02–1.72). However, this association was attenuated and non-significant after full adjustment (OR 1.16, 95% CI 0.87–1.54; Model 2; **Table 2**).

### Subgroup analysis by demographic and clinical characteristics

No statistically significant interactions were observed between sedentary time and gender, hypertension, or cardiovascular disease history (all P_interaction_ > 0.05; **Supplementary Table 2**-**S5**). In sex-stratified analyses, prolonged sedentary time (>8 h/day vs. ≤4 h/day) was significantly associated with higher odds of DKD in women (OR 2.10, 95% CI 1.27–3.47), but not in men (**Supplementary Table 6**). Sedentary behavior showed no significant association with DKD risk in the overall cohort.

### Joint analysis of leisure-time and other domain-specific physical activity on DKD risk

**Figure 1:**
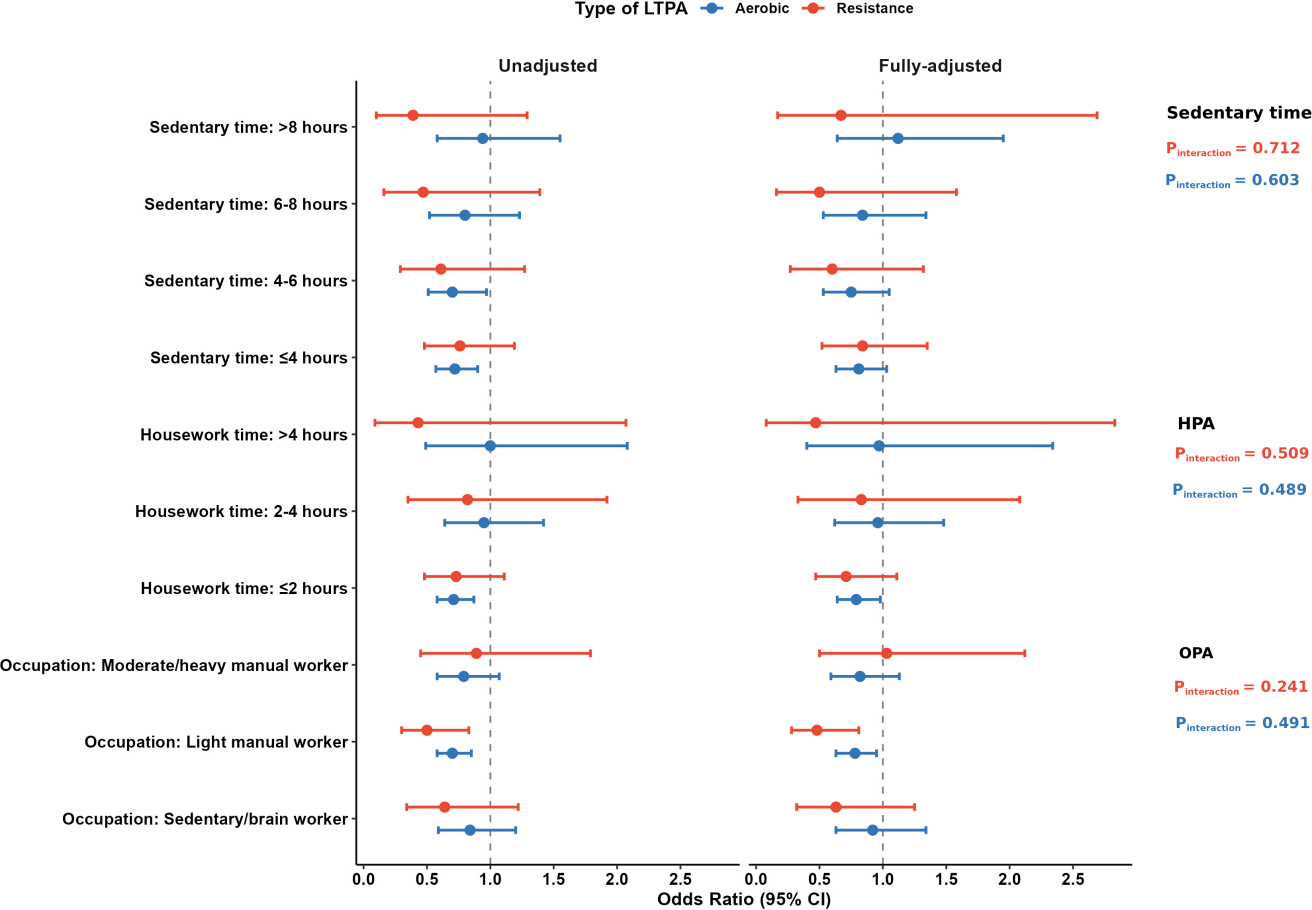
Association between leisure-time physical activity (LTPA) and diabetic kidney disease (DKD) across domains of physical activity: stratified by household, occupational, and sedentary behaviors. Full-adjusted model adjusted for gender, age, duration of diabetes, educational level, SBP, BMI, HbA1c, TG, HDL, history of hypertension, hyperlipidemia, cardiovascular disease, and medication usage. The pot and horizontal bars represent the odds ratios (ORs) and 95% CIs. AE, aerobic exercise; RE, resistance exercise; HPA, housework physical activity; OPA, occupational physical activity.

The joint analysis demonstrated that occupational physical activity, housework physical activity, and sedentary behavior potentially modified the association between LTPA and DKD risk (**Figure 1**). Among light manual workers, both aerobic (OR 0.78, 95% CI 0.63–0.95) and resistance exercise (OR 0.48, 95% CI 0.28–0.81) showed significant protective effects, whereas these benefits were attenuated in sedentary/brain workers and heavier manual workers. When stratified by housework duration, aerobic exercise was associated with reduced DKD odds only in participants reporting ≤2 hours/day of housework (OR 0.79, 95% CI 0.64–0.98), with no protective effect detected for those with 2–4 or >4 hours/day. Similarly, stratification by sedentary time revealed that in crude analyses, aerobic exercise was significantly associated with lower odds of DKD among participants with daily sedentary time ≤4 hours (OR 0.72, 95% CI 0.57–0.90) and 4–6 hours (OR 0.70, 95% CI 0.51–0.97), but not among those with sedentary time exceeding 6 hours. After full adjustment, these associations were attenuated and became borderline significant. Resistance exercise was associated with reduced odds across all sedentary time categories, although none of these reached statistical significance.

## Discussion

In this study, higher levels of leisure-time physical activity (LTPA), including both aerobic exercise (AE) and resistance exercise (RE), were associated with reduced odds of diabetic kidney disease (DKD) among individuals with diabetes. In contrast, high levels of household physical activity (HPA) or occupational physical activity (OPA) were associated with increased odds of DKD. Prolonged sedentary time was also associated with higher odds of DKD in women, but not in men. Furthermore, the protective association of LTPA with DKD might be attenuated in participants with physically demanding occupations, longer durations of housework, or greater daily sedentary time.

Partly consistent with our findings that LTPA might reduce the odds of DKD, the FinnDiane Study reported that the intensity and frequency of LTPA influenced microalbuminuria and might contribute to the development of DKD in patients with type 1 diabetes (17, 18). In addition, our results are supported by evidence that AE interventions decrease UACR (19), an early biomarker of DKD (20). However, the REACTION study reported no association between physical activity and eGFR in patients with diabetes, suggesting that physical activity may not necessarily reduce DKD risk (21). This discrepancy may be partly explained by the lack of domain-specific PA assessment in that study, in contrast to our study and others.

Of note, engaging in heavier manual labor occupations or undertaking greater amounts of housework may was associated with a lower prevalence of DKD. This finding is somewhat unexpected, as HPA or OPA are often regarded as supplementary to LTPA when the latter is insufficient (22). Our results are partly consistent with the recently proposed concept of the ‘physical activity paradox,’ which posits that LTPA improves health, whereas OPA or HPA may be detrimental (23–25). Several mechanisms may explain these findings. First, high levels of OPA or HPA are typically constant and repetitive, involving prolonged static postures and insufficient recovery periods. Continuous isometric contraction of specific muscle groups may induce chronic fatigue, while inadequate recovery can lead to sustained elevations in heart rate or blood pressure, both of which have been linked to an increased risk of kidney disease (26, 27). Second, the intensity of OPA or HPA may not meet the threshold required for health or fitness benefits (16, 28). Moreover, individuals engaged in OPA or HPA may mistakenly believe they have achieved adequate vigorous-intensity activity, leading to increased energy intake, subsequent obesity, and eventually DKD development. Finally, from a socioeconomic perspective, heavy HPA or OPA is often associated with lower socioeconomic status, which is in turn linked to unhealthy lifestyle factors such as smoking, irregular sleep patterns, and impaired psychological well-being.

In our study, longer sedentary time was not associated with increased odds of DKD in the overall population. This finding contrasts with previous reports suggesting that reducing sedentary time may improve kidney function (29), whereas prolonged sedentary behavior can contribute to kidney damage (30). Notably, we observed that prolonged sedentary time was associated with increased odds of DKD in women but not in men, suggesting a potential sex-specific difference. This aligns with prior evidence that the associations of sedentary behavior with cardiometabolic health (31), or hypertension (32), was stronger in women than in men. Several potential mechanisms may explain this sex-specific association. Women generally have lower skeletal muscle mass and may exhibit different metabolic responses to prolonged sedentary behavior, which could increase susceptibility to metabolic dysregulation and vascular dysfunction (33). In addition, hormonal factors, such as differences in estrogen-related metabolic regulation, may influence renal and cardiovascular responses to sedentary lifestyles. Behavioral factors may also play a role. For example, patterns of sedentary activities and the accuracy of self-reported sedentary time may differ between men and women, while these factors may partly contribute to the sex-specific association observed in the present study.

The strengths of this study include the use of a nationally representative sample and a comprehensive assessment of the associations between different domains of physical activity and DKD among adults with diabetes. Nevertheless, several limitations should be acknowledged. First, physical activity and sedentary behavior were self-reported rather than objectively measured, which may have introduced recall bias. Participants may have over- or under-reported their activity levels, particularly for occupational and household activities where the duration and intensity of activities may be difficult to estimate accurately. Future studies using objective measurements, such as accelerometry, may provide more precise assessments of physical activity and sedentary behavior. Second, DKD status was defined using a composite diagnostic approach incorporating macroalbuminuria, microalbuminuria with diabetic retinopathy, or microalbuminuria with long-duration type 1 diabetes. This operational definition, while aligned with clinical guidelines, may introduce heterogeneity across diagnostic pathways. Moreover, because repeated UACR measurements were not consistently available to confirm albuminuria persistence, the possibility of misclassification cannot be fully ruled out. Third, although multiple clinical covariates were adjusted for in the analyses, certain lifestyle factors, such as smoking, alcohol consumption, and detailed dietary information, were not available in the dataset. These factors may correlate with both physical activity patterns and the presence of DKD, and therefore residual confounding cannot be completely excluded. Furthermore, occupational physical activity categories were derived from job types, which may partly reflect occupational characteristics and socioeconomic conditions. Finally, the cross-sectional design precludes causal inference regarding the relationships between physical activity, sedentary behavior, and DKD. Reverse causality may exist, as individuals with DKD or more severe diabetes may reduce leisure-time exercise or shift toward less physically demanding activities.

## Conclusions

In summary, this study highlights that higher level of leisure-time physical activity, including both aerobic and resistance exercise, are associated with reduced odds of diabetic kidney disease among adults with diabetes. Conversely, higher engagement in household and occupational physical activity, particularly when coupled with prolonged sedentary behavior, may increase the odds of DKD or attenuate the protective benefits of leisure-time exercise. These findings emphasize the complexity of physical activity domains and sedentary behavior in DKD development, underscoring the importance of tailored physical activity recommendations that consider occupational demands, household responsibilities, and sedentary patterns. Future longitudinal studies incorporating objective measurements are warranted to confirm these associations and to guide effective interventions aimed at reducing DKD burden through optimized physical activity strategies.

## Data Availability

The raw data supporting the conclusions of this article will be made available by the authors, without undue reservation.
