# Supplementary material for: Associations of physical activity and sedentary time with diabetic kidney disease among adults with diabetes: a multicenter population-based study
Source: Front Endocrinol (Lausanne). 2026 Apr 22;17:1809438. doi: 10.3389/fendo.2026.1809438 (PMC13143711; doi:10.3389/fendo.2026.1809438)
Supplement: Supplementary file 1 [file Table1.pdf]

Supplementary files

Supplementary Table S1 – Physical activity questionnaire.

Supplementary Table S2 – Modes of physical activity in ChinaNeeds Study and the corresponding 2011 compendium of physical activity.

Supplementary Table S3 – Subgroup analyses for LTPA based on gender, body mass index, history of cardiovascular diseases, or history of hypertension.

Supplementary Table S4 – Subgroup analyses for HPA based on gender, body mass index, history of cardiovascular diseases, or history of hypertension.

Supplementary Table S5 – Subgroup analyses for OPA based on gender, body mass index, history of cardiovascular diseases, or history of hypertension.

Supplementary Table S6 – Subgroup analyses for daily sedentary time based on gender, body mass index, history of cardiovascular diseases, or history of hypertension.

Supplementary Table S1. Physical activity questionnaire.

Item	Question	Options
**Aerobic exercise**		
	What are the most common three forms of aerobic exercise you usually did in the previous six months?	(choose from a pre-designed list [a])
**(1)**	The most common for: XXX	
	How often did you this specific exercise per week? XXX days per week	
	How long did you spend doing this specific exercise per day? XXX hour XXX min	
**(2)**	The second most common for: XXX	
	How often did you this specific exercise per week? XXX days per week	
	How long did you spend doing this specific exercise per day? XXX hour XXX min	
**(3)**	The third most common for: XXX	
	How often did you this specific exercise per week? XXX days per week	
	How long did you spend doing this specific exercise per day? XXX hour XXX min	
**(4)**	Being unable to take exercise: Yes NO	
**(5)**	Being unwilling to take exercise: Yes NO	
**Resistance exercise**		
	What are the most common two forms of resistance exercise you usually did in the previous six months?	
**(1)**	The most common for: XXX	A. Weightlifting
	How often did you this specific exercise per week? XXX days per week	B. Dumbbells
	How long did you spend doing this specific exercise per day? XXX hour XXX min	C. Push-ups
**(2)**	The second most common for: XXX	D. Pull-ups
	How often did you this specific exercise per week? XXX days per week	E. Elastic band exercises
	How long did you spend doing this specific exercise per day? XXX hour XXX min	
**(3)**	Being unable to take exercise: Yes NO	
**(4)**	Being unwilling to take exercise: Yes NO	
**Housework physical activity**		
	Do housework activities you usually take part in (such as sweeping the floor, washing dishes, etc.)	
	How often did you this specific exercise per week? XXX days per week	
	How long did you spend doing this specific exercise per day? XXX hour XXX min	
**Occupational physical activity**		
	What occupation do you do? XXX	
	Sedentary/brain worker: such as students, office workers, coach drivers, teachers, etc.	
	Light manual worker: such as homemakers, cooks, sales staff, retirees, etc.	
	Moderate to heavy manual worker: such as such as porters, construction workers, farmers, etc.	
**Sedentary behavior**		
	How long sedentary time did you usually have? (such as watch TV, read newspapers, play poker, play chess, etc.)	
	How often did you this specific exercise per week? XXX days per week	
	How long did you spend doing this specific exercise per day? XXX hour XXX min	

[a] The list of common aerobic exercises was showed in Supplementary Table S2.

Supplementary Table S2. Modes of physical activity in ChinaNeeds Study and the corresponding 2011 compendium of physical activity.

NO.	modes of exercise	METs	2011 Compendium code
1	Walking	3.0	17165
2	Fast walking for exercise (100-120 steps/min)	4.3	17200
3	Very brisk walking (120-160 steps/min)	7.0	17230
4	Jogging	6.0	12029
5	Tai Chi/Qigong	3.0	15670
6	Square dancing	5.0	03020
7	Cycling	4.0	01010
8	Mountain climbing	6.5	17035
9	Swimmng	6.0	17035
10	Stair climbing for exercise	9.0	02065
11	Rope skipping	8.8	15552
12	Table tennis	4.0	15660
13	Badminton	5.5	15030
14	Tennis	7.3	15675
15	Basketball	6.0	15050
16	Volleyball	4.0	15710
17	Footbal	7.0	15610
18	Handball	12.0	15320
19	Gateball	3.3	15160
20	Horse riding	5.5	15370
21	Diving	7.0	18210
22	Golf	4.8	15255
23	Skiing	7.0	19075
24	Skating	7.0	19030
25	Ballroom dancing	3.0	03040
26	Gymnastics	3.8	15300
27	Jiamusi dance	4.5	03025
28	Bodybuilding exercise	3.8	15300
29	Sword dance	5.0	03020
30	Bowling	3.0	15090

Supplementary Table S3. Subgroup analyses for LTPA based on gender, body mass index, history of cardiovascular diseases, or history of hypertension.

Subgroup	Aerobic exercise meeting guideline goals		Resistance exercise meeting guideline goals	
	OR (95% CI)	$P_{interaction}$	OR (95% CI)	$P_{interaction}$
Gender		0.060		0.593
Female	0.62 (0.48, 0.80)		0.78 (0.35, 1.75)	
Male	0.83 (0.66, 1.04)		0.74 (0.49, 1.12)	
BMI, Body mass index (kg/m^2)		0.204		0.219
BMI<24 (normal)	0.68 (0.52, 0.87)		0.82 (0.48, 1.41)	
BMI≥24&<28 (overweight)	0.72 (0.55, 0.95)		0.55 (0.29, 1.05)	
BMI≥28 (obesity)	1.06 (0.71, 1.59)		1.44 (0.62, 3.32)	
History of cardiovascular diseases**		0.705		0.825
Neither CVD nor CBD	0.73 (0.60, 0.89)		0.83 (0.55, 1.25)	
CVD or CBD	0.82 (0.56, 1.18)		0.58 (0.25, 1.34)	
Both CVD and CBD	0.54 (0.23, 1.29)		0.93 (0.06, 15.41)	
Hypertension history		0.941		0.108
No	0.73 (0.56, 0.94)		0.57 (0.33, 0.99)	
Yes	0.77 (0.61, 0.96)		1.01 (0.61, 1.68)	

***Variables for adjustment:** gender, age, duration of diabetes, educational level, SBP, BMI, HbA1c,TG, HDL, history of hypertension, hyperlipidemia, cardiovascular diseases, and medication usage(except grouping factor itself).

** History of cardiovascular diseases was divided into two types of diseases belonging to cardiovascular system and cerebrovascular system

CVD: diseases of cardiovascular system, such as stenocardia, myocardial infarction, coronary atherosclerotic disease

CBD: diseases of cerebrovascular system, where patients with signs of internal carotid artery system (hemiplegia, dyskinesia, hemiblindness), or signs of vertebral basilar artery (nystagmus, ataxia, cross-over paralysis), and intracranial infarction or hemorrhage diagnosed by CT or MRI

Supplementary Table S4. Subgroup analyses for HPA based on gender, body mass index, history of cardiovascular disease or cerebrovascular disease, or history of hypertension.

Subgroup	Exposure Variable	POR	95% CI	P for trend	P interaction
Gender					0.683
Female				0.059	
	≤2h (reference)	1 (Ref)			
	>2h&≤4h	1.14	(0.86, 1.50)		
	>4h	1.52	(0.98, 2.34)		
Male				0.804	
	≤2h (reference)	1 (Ref)			
	>2h&≤4h	0.89	(0.59, 1.34)		
	>4h	1.42	(0.67, 3.00)		
BMI (kg/m^2)					0.850
<24 (normal)				0.455	
	≤2h (reference)	1 (Ref)			
	>2h&≤4h	1.08	(0.78, 1.52)		
	>4h	1.21	(0.68, 2.13)		
≥24&<28 (overweight)				0.163	
	≤2h (reference)	1 (Ref)			
	>2h&≤4h	1.02	(0.71, 1.47)		
	>4h	1.73	(0.97, 3.11)		
≥28 (obesity)				0.358	
	≤2h (reference)	1 (Ref)			
	>2h&≤4h	0.98	(0.53, 1.79)		
	>4h	1.88	(0.73, 4.83)		
History of cardiovascular diseases**					0.841
Neither CVD nor CBD				0.177	
	≤2h (reference)	1 (Ref)			
	>2h&≤4h	1.01	(0.77, 1.32)		
	>4h	1.48	(0.97, 2.25)		
CVD or CBD				0.173	
	≤2h (reference)	1 (Ref)			
	>2h&≤4h	1.16	(0.71, 1.90)		
	>4h	1.80	(0.80, 4.04)		
Both CVD and CBD				0.176	
	≤2h (reference)	1 (Ref)			
	>2h&≤4h	0.49	(0.13, 1.83)		
	>4h	1.00	(0, 0)		
Hypertension history					0.629
No				0.073	
	≤2h (reference)	1 (Ref)			
	>2h&≤4h	1.06	(0.73, 1.54)		
	>4h	1.93	(1.08, 3.45)		
Yes				0.620	
	≤2h (reference)	1 (Ref)			
	>2h&≤4h	0.99	(0.74, 1.31)		
	>4h	1.20	(0.74, 1.95)		

* Variables for adjustment: gender, age, duration of diabetes, educational level, SBP, BMI, HbA1c,TG, HDL, history of hypertension, hyperlipidemia, cardiovascular diseases, and medication usage(except grouping factor itself).

** History of cardiovascular diseases was divided into two types of diseases belonging to cardiovascular system and cerebrovascular system.

CVD: diseases of cardiovascular system, such as stenocardia, myocardial infarction, coronary atherosclerotic disease.

CBD: diseases of cerebrovascular system, where patients with signs of internal carotid artery system (hemiplegia, dyskinesia, hemiblindness), or signs of vertebral basilar artery (nystagmus, ataxia, cross-over paralysis), and intracranial infarction or hemorrhage diagnosed by CT or MRI.

Supplementary Table S5. Subgroup analyses for OPA based on gender, body mass index, history of cardiovascular diseases, or history of hypertension.

Subgroup	Exposure Variable	POR	95% CI	P for trend	P interaction
Gender					0.361
Female				0.159	
	sedentary/brain worker(reference)	1 (Ref)			
	light manual worker	1.37	(0.81, 2.32)		
	moderate or heavy manual worker	2.26	(1.28, 4.00)		
Male				0.256	
	sedentary/brain worker(reference)	1 (Ref)			
	light manual worker	1.00	(0.73, 1.37)		
	moderate or heavy manual worker	1.16	(0.83, 1.63)		
BMI (kg/m^2)					0.171
<24 (normal)				0.008	
	sedentary/brain worker(reference)	1 (Ref)			
	light manual worker	0.93	(0.62, 1.41)		
	moderate or heavy manual worker	1.82	(1.18, 2.82)		
≥24&<28 (overweight)				0.815	
	sedentary/brain worker(reference)	1 (Ref)			
	light manual worker	1.17	(0.78, 1.76)		
	moderate or heavy manual worker	1.12	(0.71, 1.79)		
≥28 (obesity)				0.978	
	sedentary/brain worker(reference)	1 (Ref)			
	light manual worker	1.52	(0.81, 2.84)		
	moderate or heavy manual worker	1.52	(0.77, 3.02)		
History of cardiovascular diseases**					0.583
Neither CVD nor CBD				0.048	
	sedentary/brain worker(reference)	1 (Ref)			
	light manual worker	1.11	(0.83, 1.48)		
	moderate or heavy manual worker	1.44	(1.05, 1.97)		
CVD or CBD				0.996	
	sedentary/brain worker(reference)	1 (Ref)			
	light manual worker	1.15	(0.62, 2.15)		
	moderate or heavy manual worker	1.83	(0.89, 3.79)		
Both CVD and CBD				0.379	
	sedentary/brain worker(reference)	1 (Ref)			
	light manual worker	0.81	(0.08, 8.19)		
	moderate or heavy manual worker	2.13	(0.15, 29.66)		
Hypertension history					0.998
No				0.121	
	sedentary/brain worker(reference)	1 (Ref)			
	light manual worker	0.89	(0.60, 1.31)		
	moderate or heavy manual worker	1.32	(0.88, 1.99)		
Yes				0.210	
	sedentary/brain worker(reference)	1 (Ref)			
	light manual worker	1.32	(0.93, 1.89)		
	moderate or heavy manual worker	1.67	(1.11, 2.51)		

* Variables for adjustment: gender, age, duration of diabetes, educational level, SBP, BMI, HbA1c,TG, HDL, history of hypertension, hyperlipidemia, cardiovascular diseases, and medication usage(except grouping factor itself).

** History of cardiovascular diseases was divided into two types of diseases belonging to cardiovascular system and cerebrovascular system.

CVD: diseases of cardiovascular system, such as stenocardia, myocardial infarction, coronary atherosclerotic disease.

CBD: diseases of cerebrovascular system, where patients with signs of internal carotid artery system (hemiplegia, dyskinesia, hemiblindness), or signs of vertebral basilar artery (nystagmus, ataxia, cross-over paralysis), and intracranial infarction or hemorrhage diagnosed by CT or MRI.

Supplementary Table S6. Subgroup analyses for daily sedentary time based on gender, body mass index, history of cardiovascular diseases, or history of hypertension.

Subgroup	Exposure Variable	POR	95% CI	$P_{\text{for trend}}$	$P_{\text{interaction}}$
Gender					0.112
Female				0.018	
	≤4h (reference)	1 (Ref)			
	4-6h	1.07	(0.78, 1.45)		
	6-8h	1.13	(0.73, 1.73)		
	>8h	2.10	(1.27, 3.47)		
Male				0.313	
	≤4h (reference)	1 (Ref)			
	4-6h	1.26	(0.96, 1.65)		
	6-8h	1.11	(0.79, 1.57)		
	>8h	1.17	(0.78, 1.75)		
BMI (kg/m^2)					0.850
<24 (normal)				0.052	
	≤4h (reference)	1 (Ref)			
	4-6h	1.19	(0.87, 1.63)		
	6-8h	1.06	(0.70, 1.60)		
	>8h	1.72	(1.08, 2.74)		
≥24&<28 (overweight)				0.526	
	≤4h (reference)	1 (Ref)			
	4-6h	1.07	(0.77, 1.47)		
	6-8h	1.08	(0.70, 1.66)		
	>8h	1.16	(0.68, 1.99)		
≥28 (obesity)				0.685	
	≤4h (reference)	1 (Ref)			
	4-6h	1.26	(0.77, 2.08)		
	6-8h	1.10	(0.57, 2.11)		
	>8h	1.10	(0.54, 2.23)		
History of cardiovascular diseases**					0.569
Neither CVD nor CBD				0.034	
	≤4h (reference)	1 (Ref)			
	4-6h	1.18	(0.93, 1.50)		
	6-8h	1.06	(0.77, 1.44)		
	>8h	1.55	(1.09, 2.22)		
CVD or CBD				0.626	
	≤4h (reference)	1 (Ref)			
	4-6h	1.02	(0.66, 1.59)		
	6-8h	1.45	(0.81, 2.61)		
	>8h	0.94	(0.46, 1.91)		
Both CVD and CBD				0.158	
	≤4h (reference)	1 (Ref)			
	4-6h	1.08	(0.37, 3.20)		
	6-8h	0.27	(0.05, 2.06)		
	>8h	0.31	(0.04, 2.67)		
Hypertension history					0.968
No				0.115	
	≤4h (reference)	1 (Ref)			
	4-6h	1.25	(0.90, 1.73)		
	6-8h	1.16	(0.77, 1.75)		
	>8h	1.43	(0.90, 2.30)		
Yes					
	≤4h (reference)	1 (Ref)		0.258	
	4-6h	1.09	(0.84, 1.41)		
	6-8h	1.07	(0.75, 1.53)		
	>8h	1.28	(0.84, 1.96)		

*Variables for adjustment: gender, age, duration of diabetes, educational level, SBP, BMI, HbA1c,TG, HDL, history of hypertension, hyperlipidemia, cardiovascular diseases, and medication usage(except grouping factor itself)
** History of cardiovascular diseases was divided into two types of diseases belonging to cardiovascular system and cerebrovascular system
CVD: diseases of cardiovascular system, such as stenocardia, myocardial infarction, coronary atherosclerotic disease
CBD: diseases of cerebrovascular system, where patients with signs of internal carotid artery system (hemiplegia, dyskinesia, hemiblindness), or signs of vertebral basilar artery (nystagmus, ataxia, cross-over paralysis), and intracranial infarction or hemorrhage diagnosed by CT or MRI
